# Chelation Combination—A Strategy to Mitigate the Neurotoxicity of Manganese, Iron, and Copper?

**DOI:** 10.3390/biom12111713

**Published:** 2022-11-18

**Authors:** Jan O. Aaseth, Valeria M. Nurchi

**Affiliations:** 1 Department of Research, Innlandet Hospital Trust, P.O. Box 104, N-2381 Brumunddal, Norway; 2 Faculty of Health and Social Sciences, Inland Norway University of Applied Sciences, P.O. Box 104, N-2418 Elverum, Norway; 3 Department of Life and Environmental Sciences, University of Cagliari, Cittadella Universitaria, 09042 Monserrato, Italy

**Keywords:** manganese, copper, iron, DMSA, deferiprone, deferasirox, p-aminosalicylate, chelating agents, neurodegenerative diseases, Wilson's disease, Parkinson's disease

## Abstract

The chelating thiol dimercaptosuccinate (DMSA) and the traditional agent D-penicillamine (PSH) are effective in enhancing the urinary excretion of copper (Cu) and lead (Pb) in poisoned individuals. However, DMSA, PSH, EDTA (ethylenediamine tetraacetate), and deferoxamine (DFOA) are water-soluble agents with limited access to the central nervous system (CNS). Strategies for mobilization of metals such as manganese (Mn), iron (Fe), and Cu from brain deposits may require the combined use of two agents: one water-soluble agent to remove circulating metal into urine, in addition to an adjuvant shuttler to facilitate the brain-to-blood mobilization. The present review discusses the chemical basis of metal chelation and the ligand exchange of metal ions. To obtain increased excretion of Mn, Cu, and Fe, early experiences showed promising results for CaEDTA, PSH, and DFOA, respectively. Recent experiments have indicated that p-amino salicylate (PAS) plus CaEDTA may be a useful combination to remove Mn from binding sites in CNS, while the deferasirox–DFOA and the tetrathiomolybdate–DMSA combinations may be preferable to promote mobilization of Fe and Cu, respectively, from the CNS. Further research is requested to explore benefits of chelator combinations.

## 1. Introduction

Acute poisonings with manganese, iron, or copper compounds can, in many cases, be satisfactorily treated by chelator monotherapy [1]. Early chelator treatment after metal exposure should preferably be administered immediately, although chelator monotherapy within 6–12 h after the intoxication is effective in many cases [1]. However, when a chelator is given several days or weeks after the initial symptoms to patients with long-term metal exposure and cerebral metal deposits, traditional chelator monotherapy has often failed. As for the metals manganese, iron, and copper, this is all the more unfortunate as cerebral accumulations of these metals have been associated with permanent and severe neurodegenerative diseases (Table 1).

*Manganese* (Mn) exposure with brain deposition can lead to a neuropsychiatric disturbance known as manganism [7] (Table 1). Its neuropsychiatric symptoms consist of mood changes and slowness of movement resembling those of Parkinson’s disease (PD) [8]. Since toxic Mn exposure occurs frequently among welders, it has been referred to as “welder’s disease”. Later reports have also documented drinking water as a source of toxic manganese exposure [2]. Current treatment of manganism is EDTA chelation; however, this treatment has shown limited efficacy [9].

*Iron* (Fe) dyshomeostasis occurs in PD patients, typically showing elevated levels of Fe bound to neuromelanin in the substantia nigra [10]. This focal siderosis in substantia nigra is apparently not accompanied by changed Fe content in other brain regions in Parkinson patients [11]. However, extracellular iron–neuromelanin complexes together with misfolded alpha-synuclein may be related to neuroinflammation in PD [3]. Other neurodegenerative diseases with brain Fe accumulation include aceruloplasminemia and Friedreich’s ataxia [12]. Excesses of Fe and Cu in nervous tissue can exert toxic effects through generation of reactive oxygen radicals (ROS) through the Fenton reaction, thus aggravating neuropsychiatric [13] and neurodegenerative diseases [4].

*Copper* (Cu) is a well-known neurotoxic metal when occurring in excess in CNS, although it is also essential for several enzymes including cytochrome c oxidase, superoxide dismutase (SOD1), dopamine beta-hydroxylase, and tyrosinase [14]. Although exogenous Cu intoxications are rare, dysregulations in the Cu metabolism may lead to Cu retention and neurological disorders, as seen in Wilson’s disease [15]. Furthermore, Cu dysmetabolism seems to play a role in the pathophysiology of Alzheimer’s disease (AD) [5], although it should be emphasized that the role of metal ions in the AD pathogenesis is still a matter of controversy [16,17]. Thus, it has been suggested that some metal transthyretin complexes provide a protective response against the AD development [18]. It has been reported that, in AD, the amount of non-ceruloplasmin Cu is increased in blood plasma compared to control values [19], which may facilitate the Cu transport into the brain [20]. Excesses of Cu and Fe have been found in the characteristic amyloid plaques of Alzheimer’s disease [21].

**Figure 1:**
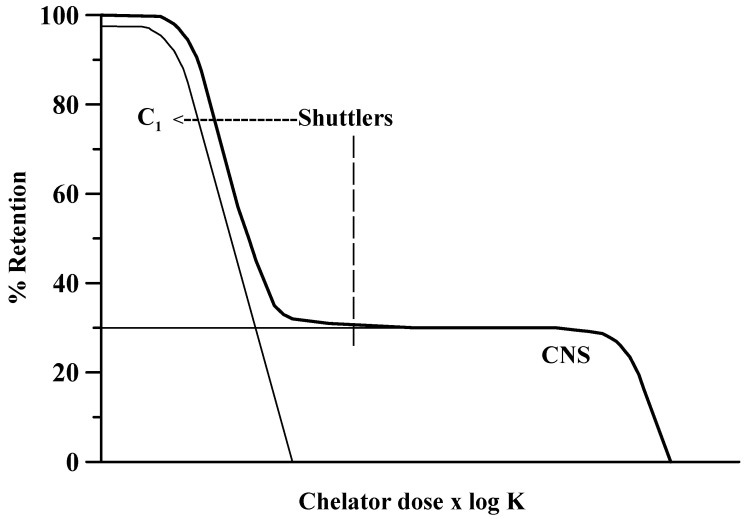
Schematic figure illustrating the hypothetic removal of a toxic metal from brain. The product of dose and stability constant (log K) of a chelator must exceed a “therapeutic threshold” to remove accumulated metal in CNS. Compartment C_1_ represents the metal level in circulating blood; the CNS compartment represents the level in the brain. The arrow indicates that a chelating shuttle can provide transfer of metal ions from CNS to C_1_, thus improving metal mobilization.

In general, significant mobilization of cerebral metal deposits in chronic diseases has not been convincingly achieved by the use of one single chelator, although monotherapy with recommended agents has shown beneficial potentials in *acute* poisonings [6,22,23,24]. The aim of the present overview is to give an update of the clinical use of chelating agents in *chronic* poisonings with cerebral deposits of manganese, iron, or copper compounds, in addition to emphasizing the presumed benefits of combining an aqueous soluble rapidly excreted chelator with a lipophilic or low-molecular-weight metal carrier with the ability to shuttle across the brain–blood barriers and, thus, deliver the toxic metal via ligand exchange to the exporting water-soluble agent (Figure 1).

## 2. Principles of Metal Chelation 

The affinity between the chelating agent and the toxic metal ion are the main qualities to take into account in chelation research and chelation therapy [25,26,27], in addition to the solubility of the chelating agent and of the formed metal chelate, both in water and in lipids [28]. Water solubility enables extracellular distribution and facilitates a fast urinary excretion, while a lipophilic chelating agent displays a higher penetration across cellular membranes, as well as across the barriers within and around the central nervous system (CNS) [1]. Consequently, the lipophilic chelators are able to exploit their function intracellularly in the brain. Diffusion of the chelating agent to the tissue deposits of metal ions is of central importance to achieve metal ion mobilization. At the same time, a fast clearance of the formed chelate, into urine if possible, is of the highest importance. Intracellular and intracerebral penetration of the chelating agent and fast urinary excretion of the chelated metal ion may be incompatible properties, and this constitutes a problem in clinical treatment. In fact, intracellular access generally needs lipophilicity, while fast renal clearance needs aqueous solubility. The major obstacle in a successful treatment of the toxicity in CNS is the blood–brain barrier (BBB), which prevents many drugs entering into the brain [29]. On the basis of these considerations, the suggestion of a therapeutic combination of two chelating agents to control this incompatibility is surely tempting [30]. 

High affinity of the chelating agent for the target toxic metal ion is critical for an efficacious chelation therapy [25]. Together, low toxicity of the agent is essential to achieve clinical use of doses high enough to mobilize the metal ion from the endogenous binding sites [31]. An a priori evaluation of the chelating properties of an agent toward the toxic metal ion can be gained from an in vitro determination of the complex formation constants [32]. Mononuclear and polynuclear complexes may form when a metal ion M reacts with a chelating agent L. The present discussion focuses on mononuclear complexes ML to exemplify the basic principles of metal chelation, even if both M and L are involved, in body fluids, in numerous side reactions. These side reactions are generally taken into account by assuming a *conditional stability constant*, defined as follows [33]:

K_cond_ = [ML]/[Mt] [Lt],

where [Mt] and [Lt] are the *total* concentrations of M and L in the studied compartment, and the mobilized fraction of the target metal ion can be evaluated as follows:

[ML]/[Mt] = K_cond_ [Lt].

The fraction of chelated metal ion, therefore, depends on the conditional constant K_cond_ and on the tissue concentration [L] of the chelating agent [31,34]. Since in vitro determined stability constants offer only elementary information [35], rigorous information on the conditional constant in vivo is hard to obtain; a chemical marker that can be used in vivo for therapeutic purposes can be obtained by observing the pM value, i.e., the negative logarithm of the free metal concentration:

pM = −log[M].

It has to be remarked that the equilibrium in vivo, e.g., in blood, is a dynamic equilibrium since ML, the formed complex, continuously disappears into urine. Therefore, an increasing pM value measured during therapy is indicative of a high efficacy of the used chelating agent. 

The HSAB theory proposed by Pearson is useful for the evaluation of chemical affinities [36,37]. For example, according to this theory, the bivalent manganese metal ion Mn^2+^ and the trivalent iron Fe^3+^ are classified as representative “hard” or oxygen-seeking metal ions (Table 2). These species have principally high affinity toward “hard” ligand groups, e.g., oxygen groups [37], such as the oxygen in EDTA. On the contrary, the bivalent iron metal ion Fe^2+^ is codified as an “intermediate” metal ion that can be chelated by nitrogen and oxygen groups [37]. Likewise, “intermediate” coordination properties are presented by the bivalent metal ions Cu^2+^ and Pb^2+^. Noticeably, in an intracellular environment, copper generally occurs in the “soft” form Cu^+^ that favors coordination to thiols such as penicillamine (PSH) or to tetrathiomolybdate [38]. In particular, vicinal dithiol groups found in the lipophilic BAL and in the water-soluble DMSA present high affinity toward “soft metal ions” such as the Cu^+^ metal ion. Here, softness is related to the deformability of the outer electron shell, as well as to the size of the ionization energy, and it involves a propensity to form covalent bonds between interacting species, which are characterized by minor differences in electronegativity. Hardness favors complexation by ionic bonds, with the binding forces being dependent upon large differences in electronegativity between interacting species. 

Animal experiments are essential for the evaluation of chelation therapy, particularly in the case of chelator combination [1]. However, extrapolation from experimental studies to clinical applications can include significant drawbacks [39], necessitating clinical studies and experiences.

**Figure 2:**
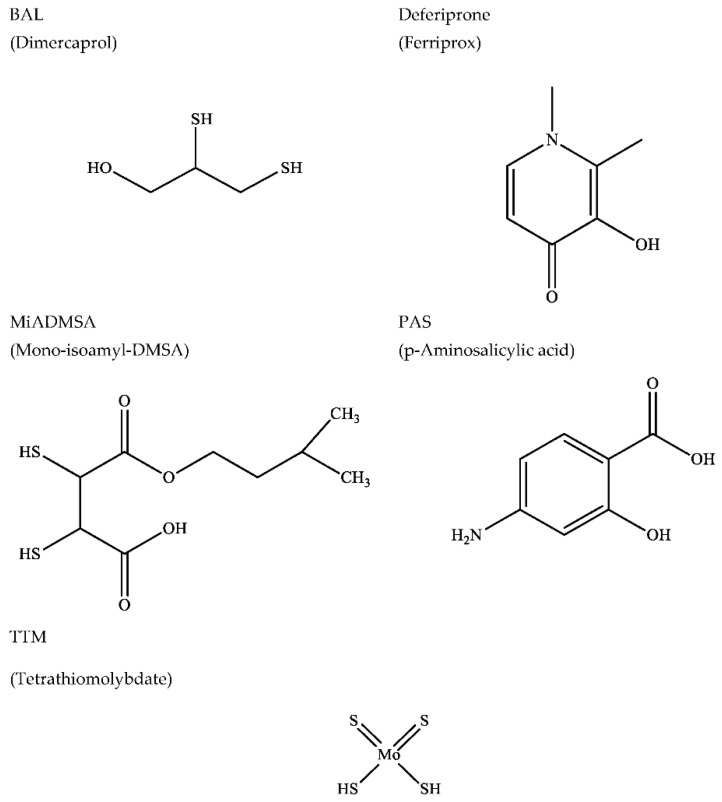
Examples of chelating agents able to penetrate across cellular membranes. Acronym, usual name, and structure.

The BAL–EDTA combined treatment previously recommended in acute lead poisoning uses rather high and often toxic BAL doses. When combined with high doses of DMSA, BAL in lower doses is supposed to act as a shuttler, since DMSA can generate a significant metal CNS-to-blood gradient through its chelation in the extracellular environment [30,40]. Beyond CaEDTA and DMSA, the extracellular chelating agents include the well-known D-penicillamine (PSH), in addition to triethylene tetramine (Trien) and deferoxamine (DFOA). Figure 2 presents a number of chelating agents that can penetrate cellular membranes to transfer metal ions along a gradient.

## 3. Chelator Combination versus Monotherapy as Therapeutic Strategy

### 3.1. Manganese Neurotoxicity and Chelation

Manganese, iron, and copper are essential trace elements for humans [41]. Occupational exposure to Mn is a primary cause of chronic Mn intoxication, usually referred to as manganism [41]. Exposure to Mn in the occupational settings occurs predominantly via inhalation, e.g., in industrial processes involving welding or smelting, since Mn is crucial for steel and stainless-steel production. Mn is also used in fungicides, e.g., in the fungicide Maneb, which, upon exposure, can result in a kind of manganism that resembles parkinsonism [42]. Manganism may also arise from intravenous Mn intake, as in long-term parenteral nutrition or in ephedron abusers who use permanganate for the synthesis of the drug [43]. Symptoms with rigidity, tremor, and bradykinesia as in manganism have also been described due to mutations in the Mn transporter gene SLC30A10 with insufficient biliary excretion of Mn [44]. Manganism, since its early descriptions around 1850, has been compared to PD. Both diseases present with rigidity, dystonia, tremor, and bradykinesia, potentially resulting in dementia. However, the neuropathology differs between these two diseases. Manganism is characterized by a loss of neurons in globus pallidus and pars reticulata of substantia nigra, whereas PD mainly affects the pars compacta [45]. The Lewy bodies characterizing advanced PD are not present in manganism [46]. Although manganism usually occurs after long-term exposure, it may appear after Mn exposure of as little as few months [47]. It is unclear why Mn affects specific brain regions such as the basal ganglia. It affects the functions of several neurotransmitters [48]. Usually, there is inflammatory activation of astrocytes and microglia during manganism [49]. Diagnosis of manganism vs. PD is typically based on exposure history and blood and urine analyses. 

Chelation therapy is the recommended treatment. The extracellularly distributed chelator CaEDTA increases the amount of Mn excreted in the urine; however, its effect on clinical symptoms has been debated [50]. The similarity of manganism to PD has led clinicians to try the amino acid L-dopa in the treatment of chronic manganese poisoning. Dopamine can also operate as a chelator [51]. *Combined chelation therapy* with CaEDTA and a membrane penetrating chelator is an attractive option for treatment of manganism. Recent experiences have reported that chelation treatment may improve the clinical features in overexposed welders when administered in the early phases of the clinical course [52]. Sodium p-aminosalicylic acid (PAS), which is presumed to act also intracellularly, has been observed to be effective in treating manganese-induced clinical manifestations in severe cases [52,53]. It is tempting to propose a combination therapy using the water-soluble agent CaEDTA together with PAS in addition to L-dopa in cases of severe manganism (Table 3).

### 3.2. Iron Neurotoxicity and Chelation

It is well known that frequent blood transfusions as given in some chronic diseases may lead to general siderosis but usually without brain Fe accumulation. Thus, the brain Fe homeostasis seems to be independent from the systemic regulation of Fe homeostasis. However, localized brain Fe deposits characterize some neurodegenerative diseases, including Friedreich’s ataxia and aceruloplasminemia [54], as well as other diseases belonging to the group of genetic disorders called neurodegeneration with brain iron accumulation (NBIAs) [54]. In addition, localized Fe deposits in substantia nigra characterize Parkinson’s disease [11,40,55,56]. A reported excess of cerebral Fe in AD seems to be limited to the amyloid plaques [57]. Physiologically, ferric transferrin in the cerebrospinal and interstitial fluid is taken up by neurons and glial cells by transferrin receptor mediated endocytosis [54].

The main iron storage protein ferritin is found in glial cells but generally not in neurons [58]. Neurons have various Fe importers, whereas ferroportin-mediated iron export ensures that intracellular levels under physiological conditions do not attain toxic levels. Neurons have several iron storage mechanisms, e.g., the endogenous chelator neuromelanin (NM), a dark-brown iron-binding pigment. In substantia nigra (SN), NM levels increase with increasing age [59], and the levels of the iron–NM complex are further increased in the early stages of PD; however, in the progression of this disease, a decrease in NM levels has been observed, although the iron levels remain increased [60]. 

Iron chelation therapy with the parenterally administered chelator deferoxamine (DFOA, Desferal) has played a vital role in the management of patients with transfusional siderosis after the introduction of this agent [61]. However, due to its primarily extracellular distribution, DFOA has limited ability to mobilize iron deposits in the CNS.

Oral treatment with the agent deferiprone (Ferriprox) offers an important supplement to the subcutaneous infusions of deferoxamine in the treatment of iron overload [62]. Deferiprone with its low molecular weight is likely to pass membranes and act intracellularly [63]. For instance, in myocardial cells deferiprone can chelate intracellular Fe deposits and reduce cardiac Fe load [64], and a similar mechanism is presumed to be responsible for mobilizing Fe deposits in the brain (Figure 1 and Table 3). Thus, deferiprone given to PD patients in a dose of 30 mg/kg/day slightly improved motor symptoms and decreased Fe content in substantia nigra after 12 months of treatment [65].

*Combined chelation therapy* with DFOA and deferiprone is an attractive option for removal of brain Fe deposits [66,67]. It was observed that combined treatment with deferoxamine and deferiprone at doses lower than normally used efficiently removed Fe from thalassemia patients, indicating a potentiation of the Fe chelation efficiency [68]. The advantages of combined chelation are in accordance with the hypothesis of increased efficacy by using two agents with different pools for chelation [1,5].

A relatively new alternative in Fe chelation therapy is deferasirox (Exjade), which is an orally active chelator forming a 2:1 complex with Fe, with the chelate being excreted via bile. Several phase III clinical trials with this agent have reported its convincing efficacy in mobilizing Fe deposits [69,70]. It is of particular interest that deferasirox can be combined with deferiprone and deferoxamine in the treatment of Fe overload [63,71]. The deferasirox–deferoxamine combination has been reported to be safe and efficacious [72]. Deferasirox when combined with DFOA appears to be associated with reduction in cerebral Fe overload and reduced progression of iron-induced neurodegeneration [73].

### 3.3. Copper Neurotoxicity and Chelation

In exogenous overexposure, as well as in the metabolic error known as Wilson’s disease, copper can exert toxic effects. Copper compounds are used in wood preservatives and fungicides, as pigments in paints, and as additives to fertilizers. Numerous fungicides still have Cu in their formulations [74]. As for Cu in drinking water, there are large differences between countries, especially depending on the use of Cu pipes in the water supply [75]. Significant Cu exposure has been recorded in Cu smelter workers and in workers in Cu refineries [76]. It has been suggested that copper dysmetabolism may play a role for the progression of AD [77,78] since the extra-neuronal plaques containing amyloid-beta peptide (Aβ) have been found to be enriched with Cu. However, the role of copper and iron in the progression of AD is still a matter of debate [16,17]. It has been claimed that anti-copper therapy may affect positively a certain percentage of AD patients [79] in agreement with the alleged existence of different subtypes of AD [80]. Here, it is relevant that benefits have been observed by lipoic acid treatment [81], as well as by selenium supplementation [82], since these therapies possess copper antagonistic properties.

With regard to disturbances in the copper metabolism, Wilson disease (WD) is known as an autosomal recessive disease affecting primarily the Cu excretion via bile and the hepatic synthesis of ceruloplasmin [83] caused by mutation in the ATP7B gene [84,85]. Defective copper excretion leads to a gradual accumulation of Cu in the liver and subsequently in brain and other organs. In the brain, most copper is selectively deposited in the basal ganglia, particularly in the putamen and globus palliidus. The traditional view on the pathophysiology of neurologic symptoms in WD holds that overwhelming of the liver storage capacity for Cu leads to its spillage into plasma and subsequently into cerebrospinal fluid, leading to cellular uptake and oxidative damage in the CNS [85]. ATP7B is also expressed in the brain, especially in the choroid plexus and basal ganglia [86], and the cerebral copper deposition in WD may be accentuated by ATP7B dysfunction in CNS. The classical studies by Walshe [87] demonstrated the effect of D-penicillamine in Wilson’s disease. The primary distribution volume of penicillamine is the extracellular space, although some membrane transfer of its Cu chelates may occur [88]. Unfortunately, PSH may give rise to adverse effects [89], the most serious being immune reactions leading, e.g., to immune complex nephritis. Furthermore, penicillamine may exacerbate neurological symptoms in the initial phase of treatment [90]. Another water-soluble chelator, triethylene tetramine (Trien), has been recommended as an alternative drug [91]. Tetrathiomolybdate (TTM), given either as its ammonium salt or as a bis-choline formulation [92], may represent a better alternative, as it facilitates biliary Cu excretion, and TTM can also, to some extent, cross the BBB [93]. As for another chelator, namely, dimercaptosuccinic acid (DMSA), there is substantial experience with this drug as a copper antidote in China [94], although it is still insufficiently evaluated in Wilson’s disease patients in the Western world [95]. Combination therapy using DMSA and TTM, with the latter given at low doses, appears to represent a promising alternative in neurological Wilson’s disease [23]. TTM passes cellular membranes via a glutathione-mediated process and may, thus, function as a brain-to-blood shuttler for Cu mobilization [23], while the extracellularly distributed DMSA promotes urinary excretion.

## 4. Summary and Perspectives

Today, CaEDTA is a standard treatment for manganese poisonings, DFOA *or* deferiprone is recommended for treatment of siderosis, and DMSA is considered promising for poisonings with copper although PSH is the traditional agent in cases of WD. It should be recalled here that adverse effects of PSH are so common as to be therapy-limiting in several cases. In the treatment of metal poisonings with cerebral deposits and neurological symptoms, a strategy with combined chelation therapy using a lipophilic in addition to a hydrophilic chelator may improve the therapeutic value. A lipophilic or low-molecular-weight agent tentatively binds and transfers the toxic metal from intracellular binding sites along a concentration gradient from brain to the circulation. Subsequently, after ligand exchange, metal excretion is taken care of by a hydrophilic chelator, e.g., by EDTA, DFOA, or DMSA. A simplified model illustrating the problems with mobilization of toxic metals from CNS is shown in Figure 1. This model illustrates that both the stability constant expressed by log K and the dose of the chelator expressed by [L] have to be high to reduce deposits in the brain. However, mobilization across biological barriers may be facilitated by combining a brain-to-blood shuttling agent with a water-soluble chelator for decorporation, e.g., PAS–EDTA, deferasirox–DFOA, or TTM–DMSA combinations, depending on the toxic metal in question. Such a combined regimen is proposed for treatment in *subacute or chronic cases* in the present review. The “shuttling plus decorporation” principle has to some extent already been adopted for mobilization of Fe in thalassemia and related sideroses by using the extracellularly distributed DFOA in combination with the low-molecular-weight agent deferiprone. Manganism may require a triple combination with CaEDTA, PAS, and L-dopa. Further exploration of the combined chelator concept to target intracerebral and intracellular metal toxicity is requested, both experimentally and clinically.

Here, it should be admitted that currently available chelators are not as target-specific as endogenous chaperones for the metals in question. Upon designing new metal-binding drugs, the targeting of intracellular sites such as the mitochondria represents a challenge. For instance, currently available chelators seem inefficient in mobilizing metal deposits in mitochondria [24] that are characteristic iron deposition sites in Friedreich’s disease [96]. Developing target-specific drugs for mobilization will be a research priority in the future. In addition, chronotherapy might become a pharmacotherapeutic option, as new evidence indicates that the blood–brain barrier is more permeable at night than during the day, and brain clearance appears to be highest at night [23].

Today, combination therapy represents an attractive approach for enhancing metal mobilization from the body by reducing individual doses of chelators, thereby limiting drug-specific adverse reactions. Thus, combination therapy with two or more drugs may prove more efficient and tolerable than monotherapy [97]. Regarding copper mobilization from the brain, lipoic acid and TTM can cross the blood–brain barrier and, thus, act as brain-to-blood shuttlers [98]. In blood a hydrophilic drug, e.g., Trien or DMSA, may efficiently serve to promote urinary excretion.

Cancer therapy has shown great interest in antagonizing iron and copper effects in tumors to limit angiogenesis, as well as for the delivery of extra metal in order to enhance production of cytotoxic ROS (reactive oxygen species) in malignant cells. Thus, the studies on chelator effects and metal antagonism can benefit from the cancer research field and vice versa [99,100].

Crucial therapeutic principles, which are valid for the treatment of Mn, Fe, and Cu toxicity, can be summarized as follows:

(1) Symptoms on neurotoxicity should ideally be identified early and chelation therapy should ideally be started before substantial irreversible brain damage has occurred. However, although symptoms on neurodegenerative diseases cannot be reversed, it will be of great interest if the disease progression can be prevented.
(2) Doses and circulating chelator levels must be controlled to enhance intended metal removal.
(3) The levels of essential metals must remain sufficient within cells for metalloenzyme biosynthesis.
(4) Regular control of blood and tissue metal pools is needed.

Patients on chelation therapy need a close clinical follow-up and neurological assessment plus monitoring of renal and liver functions. In treated cases of Wilson’s disease, as well as in NBIAs and in manganism, collection of genetic information is essential to establish valid genotype–phenotype information. Conclusively, it should be emphasized that there is a lack of high-quality evidence to estimate the relative therapeutic effects of drugs and drug combinations used in the medical conditions under investigation. Multicenter randomized comparative trials are highly needed.

## Figures and Tables

**Table 1:** Neurodegenerative diseases associated with cerebral metal depositions.

Disease	Metal Deposition
Manganism	Long-term manganese exposure [2]
Parkinson’s disease	Cerebral iron deposition in substantia nigra [3]
Aceruloplasminemia	Cerebral iron deposition [4]
Neurologic Wilson’s disease	Cerebral copper deposition [5]
Alzheimer’s disease	Associated with copper deposits in brain [6]

**Table 2:** Codification of metal ions and of coordinating groups, according to their hard, soft, or intermediate nature, in agreement with the HSAB theory of Pearson [36,37].

Metal Ions			Coordinating Groups		
Hard (Oxygen-Seekers)	Intermediate	Soft	Hard	Intermediate	Soft
Mg^2+^, Ca^2+^, Sr^2+^, Mn^2+^, Cr^3+^, Fe^3+^	Pb^2+^, Fe^2+^, Cu^2+^	Hg^2+^, CH_3_Hg^+^, Cu^+^	OH^−^, RCOO^−^, RO^−^	RNH_2_	RSH, RS^−^, R-Se^−^

**Table 3:** Proposed chelator combinations (for decorporation plus a shuttling agent) in cerebral depositions of manganese (manganism), iron (NBIAs, see text), or copper (i.e., Wilson’s disease).

Disease	Chelator Combination
Manganism	CaEDTA + PAS
Cerebral iron deposition	DFOA + deferiprone, or DFOA + deferasirox
Neurologic Wilson’s disease	DMSA + TTM, or Trien + TTM
